# Aortic Arch Mural Thrombus Concurrent with Deep Surgical-Site Infection after Colorectal Cancer Surgery in an Enhanced Recovery after Surgery Program: A Case Report

**DOI:** 10.70352/scrj.cr.25-0772

**Published:** 2026-02-28

**Authors:** Shuto Nakashima, Yoshiaki Fujimoto, Takuya Honboh, Kosuke Hirose, Taichi Nagano, Huanlin Wang, Jun Okadome, Fumihiko Hirai, Noboru Harada, Seiya Kato, Hiroyuki Ito, Noriaki Sadanaga, Tomoharu Yoshizumi

**Affiliations:** 1 Department of Surgery, Saiseikai Fukuoka General Hospital, Fukuoka, Fukuoka, Japan; 2 Department of Pathology, Saiseikai Fukuoka General Hospital, Fukuoka, Fukuoka, Japan; 3 Department of Surgery and Science, Graduate School of Medical Sciences, Kyushu University, Fukuoka, Fukuoka, Japan

**Keywords:** aortic arch mural thrombus, hypercoagulability, postoperative surgical-site infection, enhanced recovery after surgery, nonbacterial thrombotic endocarditis, *Streptococcus anginosus*, colorectal surgery

## Abstract

**INTRODUCTION:**

Aortic mural thrombus (AMT) in a non-atherosclerotic aorta is rare but potentially catastrophic and may be difficult to distinguish from septic aortic pathology when it occurs alongside a deep postoperative infection. Enhanced recovery after surgery (ERAS) shortens hospital stay and shifts the recognition of serious complications to the early post-discharge period. We report the case of a patient who underwent colorectal cancer surgery within an ERAS protocol who developed a large AMT on POD 10, coincident with *Streptococcus anginosus-*positive deep surgical-site infection (SSI) but without bacteremia or aortitis on imaging.

**CASE PRESENTATION:**

A 76-year-old male with stage IVc cecal adenocarcinoma and diabetes underwent robotic-assisted ileocecal resection via the ERAS pathway. Prophylactic cefmetazole was discontinued within 24 h, and the patient was discharged on POD 5 with down-trending but elevated C-reactive protein levels. On POD 10, the patient presented with fever, leukocytosis, and decreased mobility. Contrast-enhanced CT revealed a ~38-mm AMT without mural thickening, abnormal enhancement, periaortic fat stranding, aneurysmal dilatation, or complex atherosclerotic plaque, in addition to deep port-site infection and intra-abdominal abscesses. Blood cultures (two sets) remained negative, whereas abscess and wound cultures yielded *S. anginosus* with polymicrobial co-pathogens. The patient underwent surgical washout and drainage, broad-spectrum antibiotics (piperacillin-tazobactam, followed by ceftriaxone and metronidazole), and systemic anticoagulation with unfractionated heparin. Transesophageal echocardiography showed a mural arch mass corresponding to the CT lesion, but no definite valvular vegetation or new significant regurgitation. On POD 16, the patient developed acute left common–internal carotid occlusion with a large middle cerebral artery infarction and died on POD 20 of septic shock and disseminated intravascular coagulation.

**CONCLUSIONS:**

In this patient who underwent ERAS colorectal cancer surgery, AMT developed around POD 10 in parallel with SAG-positive deep SSI, but without aortitis or bacteremia, favoring a bland mural thrombus driven by malignancy- and sepsis-related hypercoagulability while retaining nonbacterial thrombotic endocarditis/infective endocarditis in the differential diagnosis. The case highlights PODs 7–10 as a vulnerable window in ERAS pathways and supports a focused safety bundle that includes CRP-guided discharge thresholds, selective low-dose imaging, and POD 7 ± 1 follow-up to improve early post-discharge surveillance.

## Abbreviations

AMT
: aortic mural thrombus
ERAS
: enhanced recovery after surgery
ICA
: internal carotid artery
IE
: infective endocarditis
LLQ
: left lower quadrant
MCA
: middle cerebral artery
NBTE
: nonbacterial thrombotic endocarditis
NET
: neutrophil extracellular trap
SAG
: *Streptococcus anginosus* group
SSI
: surgical-site infection
TEE
: transesophageal echocardiography
VTE
: venous thromboembolism

## INTRODUCTION

ERAS reduces postoperative morbidity and length of stay in colorectal surgery, but shifts complication recognition toward an earlier post-discharge window.^1)^ In a large cohort of more than 21000 colorectal cancer resections, nearly one-third of postoperative venous thromboembolic events were diagnosed after discharge, with a median time to diagnosis of 9 days (interquartile range: 4–16 days), indicating that serious thrombotic complications frequently cluster within the first 2 postoperative weeks.^2)^ Therefore, tailoring surveillance and safety net strategies in the early post-discharge period is becoming increasingly important in colorectal oncology.

Among the vascular events that occur during this interval, AMT is uncommon but potentially catastrophic; disturbed flow in the aortic arch promotes thrombus formation and embolization.^3)^ Differentiating bland AMT from infective pathology is critical. Infective aortitis or mycotic aneurysm typically shows CT findings such as mural thickening, enhancement, and periaortic fat stranding, whereas bland AMT may lack these features and often occur without bacteremia.^4)^

SAG is strongly associated with deep abscesses and can markedly amplify local and systemic inflammation in intra-abdominal infections, which may contribute to a prothrombotic milieu in susceptible hosts.^5)^ In parallel, malignancy-related NBTE causes sterile valvular vegetation and embolic phenomena despite negative blood cultures, and remains an important differential diagnosis in cancer patients with arterial events.^6)^

We report the case of a patient with ERAS colorectal cancer surgery who developed a large AMT on POD 10, coincident with SAG-positive deep SSI, but without bacteremia or aortitis on imaging. What this case adds is an ERAS-phase constellation rarely described—POD 10 AMT with SAG-positive deep SSI in the absence of aortitis and bacteremia—that supports a pragmatic diagnostic pathway favoring bland thrombus (while retaining NBTE/IE in the differential diagnosis) and defines ERAS safety-net triggers (CRP-guided discharge, selective low-dose imaging, review on POD 7 ± 1).

## CASE PRESENTATION

### Patient and comorbidities

The patient, a 76-year-old male, presented with stage IVc cecal adenocarcinoma and type 2 diabetes (HbA1c, 7.6%); he had received no prior systemic anticancer therapy.

### Index operation (POD 0) and indication

Semi-elective surgery was performed for malignant large-bowel obstruction and appendicitis secondary to the primary tumor. The patient underwent robotic-assisted ileocecal resection via the ERAS pathway. Intraoperatively, the tumor infiltrated the retroperitoneum with partial rupture/perforation. The terminal ileum was edematous and erythematous with an inflammatory obstruction, and appendiceal abscesses were absent. Prophylactic cefmetazole treatment was discontinued within 24 h.

### Early postoperative course (POD 1–5)

**Fig. 1:**
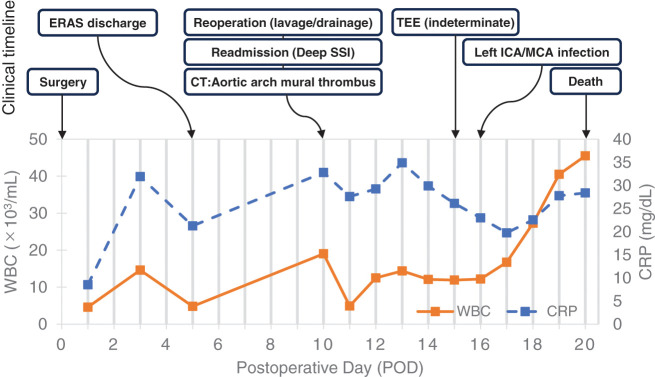
Clinical timeline from PODs 0–20. The upper panel depicts key clinical events, including ERAS-based discharge on POD 5, readmission on POD 10 with deep surgical-site infection on POD 10, detection of an aortic arch mural thrombus on contrast-enhanced CT, and subsequent development of a left internal carotid artery/middle cerebral artery infarction on POD 16. The lower panel shows serial changes in white blood cell count and C-reactive protein levels over the same period.

The patient remained afebrile (<37°C). Laboratory trends were as follows: WBC 4.6→14.6→4.8 ×10³/μL; CRP 8.52→31.9→21.3 mg/dL (PODs 1→3→5), indicating a peak on POD 3 followed by a downward trend. Clinical improvement was observed, without wound contamination, abdominal pain, or signs of SSI. Pharmacological VTE prophylaxis with enoxaparin 2000 IU (20 mg) was administered subcutaneously twice daily on PODs 2–5. No intra-abdominal drain was placed, in accordance with our institutional policy of not routinely placing drains after right hemicolectomy. He was discharged on POD 5, tolerating a regular diet with restored bowel function and presenting a decreasing CRP level, while remaining clinically stable. The overall clinical trajectory from PODs 0 to 20 is illustrated in **Fig. 1**.

### Readmission (POD 10)

On POD 10, the patient was readmitted due to fever and decreased mobility. Vital signs were as follows: blood pressure, 126/60 mmHg; heart rate, 135 beats per minute, SpO^2^, 98% on room air; and Japan Coma Scale score of 0. The LLQ port site had a scant purulent exudate. Laboratory findings showed WBC 19 × 10³/μL and CRP 32.8 mg/dL.

### Imaging (POD 10)

**Fig. 2:**
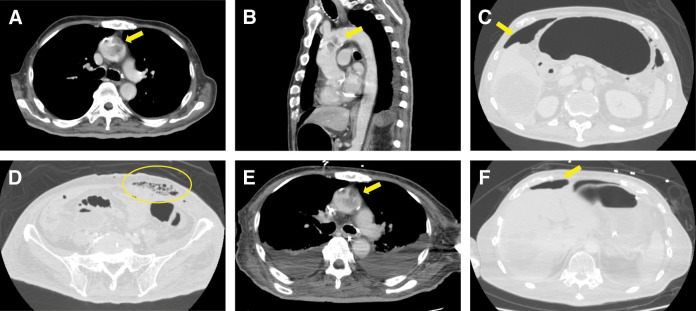
Contrast-enhanced CT findings of aortic arch mural thrombus without aortitis and concomitant deep port-site infection. On POD 10, axial and sagittal images of the aortic arch (**A**, **B**) show a coarse mural filling defect consistent with a large thrombus (arrows), without associated aortic wall thickening, abnormal mural enhancement, periaortic fat stranding, aneurysmal dilatation, or complex atherosclerotic plaque. Abdominal CT on POD 10 (**C**, **D**) demonstrates deep port-site infection, with gas tracking along the fascial plane and encapsulated fluid collections (contour line and arrows). Follow-up CT on POD 16 (**E**, **F**) shows a slightly smaller but persistent aortic arch thrombus and decreased intraperitoneal free gas (arrows), in line with partial resolution of the intra-abdominal septic focus.

Contrast-enhanced CT (**Fig. 2A**–**2D**) showed a coarse mural filling defect in the aortic arch (~38 mm) without associated aortic wall thickening, abnormal mural enhancement, periaortic fat stranding, aneurysmal dilatation, or complex atherosclerotic plaque. The abdomen demonstrated an LLQ port-site fascial infection with gas, encapsulated collections at the LLQ port site and right paracolic gutter, and diffuse extraluminal gas, consistent with a deep SSI and intra-abdominal abscesses. No definite anastomotic leakage was observed.

### Reoperation (POD 10)

The patient underwent open abdominal lavage and drainage. Turbid ascites and abscess formation were widely distributed from the right abdomen to the pelvis along the previous surgical field. The ileocolic anastomosis was intact, with no evidence of anastomotic leakage or bowel perforation. An abscess was also identified around the LLQ port site, consistent with a deep SSI, and was considered the primary source with secondary intraperitoneal spread, in accordance with the preoperative CT findings.

Three drains were placed toward the right subphrenic space, pelvic cavity, and left subphrenic space. Because the LLQ port site infection was associated with extensive gas-forming fasciitis, a cruciate incision of the anterior rectus sheath was additionally performed.

### Microbiology (pre-antibiotic change, POD 10)

Two sets of blood cultures (arterial and venous) were negative.

Abscess cultures (semiquantitative) yielded: SAG 3+; *Klebsiella oxytoca* 1+, scant growth of *Pseudomonas aeruginosa*, and presence of *Klebsiella pneumoniae*, *Bacteroides fragilis*, and *Parvimonas micra*.

Wound cultures showed scant growth of *P. aeruginosa*, and presence of *K. pneumoniae*, and *Enterococcus casseliflavus*.

### Management (POD 10 onward)

Broad-spectrum antibiotics were adjusted to 3 g piperacillin-tazobactam every 8 h (PODs 10–16), followed by 1 g ceftriaxone every 12 h and 500 mg metronidazole every 8 h (PODs 17–20). Unfractionated heparin 9600 IU/day was administered on PODs 10–18. The patient required ICU care with mechanical ventilation (PODs 10–20) and continuous hemodiafiltration (PODs 12–19) for oliguric renal failure.

### Inflammatory trend

The WBC count (×10³/μL) evolved as follows: 19→12.5→14.4→12.1→11.9→12.2→16.7→27.3→40.5→45.5 (PODs 10–19). CRP (mg/dL) changed as follows: 32.8→29.2→34.9→29.9→26.1→23.0→19.7→22.5→27.8→28.3.

### Post-reoperation course

Following reoperation, the intra-abdominal infection showed a favorable response to surgical drainage and antimicrobial therapy, with an initial improvement in inflammatory markers and resolution of systemic inflammatory signs.

No evidence of persistent or new intra-abdominal abscess formation was observed on follow-up imaging.

Blood cultures remained negative, and no other infectious foci, including pneumonia, urinary tract infection, or catheter-related infection, were identified during the subsequent clinical course.

However, despite this initial response, systemic inflammation later worsened during the clinical course, preceding the patient’s deterioration.

### Cardiovascular assessment and events

TEE revealed a mural mass in the aortic arch corresponding to the CT lesion but no definite valvular vegetation or leaflet thickening. No typical oscillating masses were observed on the aortic or mitral valves and no new significant valvular regurgitation was observed. Overall, this study did not provide clear evidence of IE or NBTE.

On POD 16, the patient developed an acute left common ICA occlusion with a large left MCA infarction and a midline shift. Follow-up CT on POD 16 (**Fig. 2E** and **2F**) showed that the aortic thrombus was slightly smaller but persistent, with decreasing free gas, ascites, and peritoneal thickening consistent with peritonitis.

### Outcome

Despite initial local control of the intra-abdominal infection after reoperation, the patient experienced progressive systemic deterioration, even despite escalation of antimicrobial, anticoagulation, and organ-supportive therapies. He died on POD 20 from septic shock and disseminated intravascular coagulation. An autopsy was not performed, according to the family's wishes.

## DISCUSSION

In this ERAS oncology case, the patient developed an AMT in the early post-discharge window, contemporaneous with a SAG-positive deep SSI, and without bacteremia or aortitis on imaging. ERAS reduces morbidity and length of stay; however, when applied to colorectal oncology, it may shift recognition of serious complications to the first 1–2 weeks after discharge, a period in which large registry data show that postoperative thrombotic events frequently cluster around PODs 7–10.^1,2)^ This pattern supports the need for an explicit safety network for high-risk patients. Large database analyses have shown that a meaningful proportion of postoperative VTE events are diagnosed after discharge, and have identified patient- and procedure-related factors associated with post-discharge VTE (e.g., advanced malignancy, obesity, steroid use, open surgery, and prolonged hospitalization).^7)^ Importantly, these findings indicate that thrombotic risk may still be present even in the absence of some traditional risk factors, underscoring the need for individualized risk assessment and risk-stratified post-discharge surveillance. Likewise, postoperative inflammatory markers can help identify patients at higher risk of infectious complications after colorectal surgery; several studies have reported that postoperative CRP measurement (particularly around PODs 3–5) has clinical utility for predicting or ruling out infective complications including anastomotic leak, which can inform discharge planning and post-discharge surveillance.^8)^ In this case, although the CRP level remained elevated at discharge, it had already peaked and showed a clear downward trend, and the patient met the other clinical discharge criteria under the ERAS protocol, including the absence of fever, stable abdominal findings, and recovery of gastrointestinal function. Nevertheless, this case suggests that patients with locally advanced colorectal cancer accompanied by inflammatory features may require more cautious postoperative monitoring, even when laboratory parameters appear to be improving. However, continued inpatient observation was also considered reasonable given the locally advanced disease with inflammatory features, but discharge was ultimately chosen in accordance with the patient’s strong preference after shared decision-making.

The key diagnostic question is whether an identified AMT is septic or bland (**Table 1**). Infective aortitis or mycotic aneurysms typically show mural thickening, enhancement, and periaortic stranding on CT and are often bacteremic.^4)^ In our patient, the aortic arch had a non-aneurysmal contour without ulcerated or complex atherosclerotic plaque or mural thickening, abnormal enhancement, or periaortic fat stranding, and serial blood cultures remained negative—findings that favor bland AMT in an apparently non-atherosclerotic aorta driven by hypercoagulability rather than overt aortic wall infection.^3,4)^

**Table 1:** Key discriminators among septic aortic thrombosis, bland mural thrombus, non-bacterial thrombotic endocarditis (NBTE), and infective endocarditis (IE), with this case mapped across domains.

Feature	Septic aortic thrombosis	Bland mural thrombus	NBTE	IE	This case
CT findings (wall thickening/enhancement/periaortic fat stranding)	○	○	▲	×	▲
Blood culture	○	×	×	○	×
Identification of infection source	○	×	×	○	×
TEE findings	○	▲	×	○	▲
Inflammatory markers	○	▲	▲	○	▲
Clinical course (response of thrombus)	▲	○	×	○	○
Pathology/Autopsy (NA)	×	○	×	×	○

The table summarizes the clinical, laboratory, imaging, microbiological, and embolic pattern features that help distinguish septic aortic thrombosis from bland mural thrombus, NBTE, and IE. A circle (○) indicates a typical or strongly supportive feature for a given entity, a cross (×) indicates that the feature is generally absent, and a triangle (▲) indicates a possible but non-characteristic finding. In the “This case” column, these symbols denote whether each discriminator was present, absent, or only partially fulfilled in this patient.

Cancer and sepsis drive hypercoagulability through tissue factor upregulation, endothelial injury, platelet activation, and NET formation, predisposing patients to arterial thrombosis even without plaque rupture.^9,10)^ Flow separation in the aortic arch increases the embolic risk, which is consistent with subsequent carotid occlusion and MCA infarction.^3,9)^

NBTE remains a relevant differential diagnosis for malignancies with arterial events and negative cultures.^6)^ In this patient, TEE did not reveal discrete valvular vegetations, typical oscillating masses, or new significant valvular regurgitation, making clinically overt IE or NBTE unlikely. However, given the single-timepoint assessment, advanced malignancy, and profound systemic inflammation, small sterile valvular thrombi below the resolution of echocardiography could not be completely excluded.

SAG commonly causes intra-abdominal abscesses and is associated with marked local and systemic inflammatory responses. In this setting, SAG-positive deep SSI is more plausibly viewed as one of several triggers of sepsis-induced coagulopathy rather than a direct cause of thrombosis, potentially contributing to a prothrombotic state in combination with underlying malignancy and postoperative stress.^5,10)^

Management of non-atherosclerotic AMT is individualized: anticoagulation alone yields resolution in many series, while mobile thrombi, recurrent emboli, or anatomy at risk may justify endovascular or surgical intervention.^11–13)^ In our case, rapid neurological deterioration and multiorgan failure precluded the consideration of invasive arch interventions.

### Practice implications

For high-risk ERAS colorectal cases, our experience suggests the following measures:

(i) Recognizing approximately PODs 7–10 as a potentially vulnerable period for serious complications and providing explicit return precautions and structured follow-up in this interval, as suggested by prior thromboembolic timing data and the present case.^1,2)^
(ii) When CT shows a mural thrombus without aortitis and cultures are negative, prioritizing bland AMT while reassessing for NBTE.^3,4,6)^
(iii) Combining rigorous source control and targeted antibiotics for SAG-positive SSI with optimized anticoagulation.^5,11–13)^

### Limitations

A lack of autopsy prevented pathological confirmation of the thrombus type and NBTE; nonetheless, imaging, cultures, and clinical course support a bland AMT with NBTE in the differential diagnosis.

## CONCLUSIONS

In our patient with ERAS colorectal cancer surgery, a POD 10 AMT occurred alongside a SAG-positive deep SSI without aortitis on CT and with negative blood cultures, supporting a bland-first interpretation driven by malignancy- and sepsis-related hypercoagulability; NBTE/IE was retained in the differential diagnosis. Recognizing approximately PODs 7–10 as a potentially vulnerable window, as suggested by prior data and this case, a focused safety bundle, comprising CRP-guided discharge thresholds, selective low-dose imaging, review on POD 7 ± 1, and individualized anticoagulation with rigorous source control, offers practical steps to reduce similar events in high-risk ERAS cases.

## DECLARATIONS

### Availability of data and materials

All data supporting the conclusions of this study have been included in the article.
